# Procalcitonin, lipopolysaccharide-binding protein, interleukin-6 and C-reactive protein in community-acquired infections and sepsis: a prospective study

**DOI:** 10.1186/cc4866

**Published:** 2006-03-28

**Authors:** Shahin Gaïni, Ole Græsbøll Koldkjær, Court Pedersen, Svend Stenvang Pedersen

**Affiliations:** 1 Department of Infectious Diseases, Odense University Hospital, Odense, Denmark; 2 Department of Clinical Biochemistry, Sønderborg Hospital, Sønderborg, Denmark

## Abstract

**Introduction:**

Clinicians are in need of better diagnostic markers in diagnosing infections and sepsis. We studied the ability of procalcitonin, lipopolysaccharide-binding protein, IL-6 and C-reactive protein to identify patients with infection and sepsis.

**Methods:**

Plasma and serum samples were obtained on admission from patients with suspected community-acquired infections and sepsis. Procalcitonin was measured with a time-resolved amplified cryptate emission technology assay. Lipopolysaccharide-binding protein and IL-6 were measured with a chemiluminescent immunometric assay.

**Results:**

Of 194 included patients, 106 had either infection without systemic inflammatory response syndrome or sepsis. Infected patients had significantly elevated levels of procalcitonin, lipopolysaccharide-binding protein, C-reactive protein and IL-6 compared with noninfected patients (*P *< 0.001). In a receiver-operating characteristic curve analysis, C-reactive protein and IL-6 performed best in distinguishing between noninfected and infected patients, with an area under the curve larger than 0.82 (*P *< 0.05). IL-6, lipopolysaccharide-binding protein and C-reactive protein performed best in distinguishing between systemic inflammatory response syndrome and sepsis, with an area under the curve larger than 0.84 (*P *< 0.01). Procalcitonin performed best in distinguishing between sepsis and severe sepsis, with an area under the curve of 0.74 (*P *< 0.01).

**Conclusion:**

C-reactive protein, IL-6 and lipopolysaccharide-binding protein appear to be superior to procalcitonin as diagnostic markers for infection and sepsis in patients admitted to a Department of Internal Medicine. Procalcitonin appears to be superior as a severity marker.

## Introduction

Sepsis is a common condition affecting an increasing number of hospitalized patients [1]. The prevalence of severe sepsis among inpatients varies between 2% and 11% [2]. Sepsis can be difficult to distinguish from other conditions causing systemic inflammatory response syndrome (SIRS) [3,4]. For the appropriate management of patients presenting with SIRS it is important to be able to distinguish between infectious and noninfectious causes as early as possible. This might help identify patients who need antibiotic treatment and help to avoid using antibiotics in those without infection.

C-reactive protein (CRP) has been used as a marker of infection for many years. Elevated CRP levels are seen in infection, in autoimmune disease, in cancer, in trauma and in surgery [5]. Other markers have recently been introduced as possible candidates for use in clinical practice. Procalcitonin (PCT) is a protein that has been proposed as a sensitive and specific marker of sepsis. Elevated levels of PCT have been associated with severe bacterial infections among children and adults [6]. Contrary to most other markers evaluated in the past, PCT has been reported to be specific in discriminating between viral infection and bacterial sepsis [7]. The origin and biological function of PCT in severe infection is not clarified.

Lipopolysaccharide-binding protein (LBP) is an acute-phase protein that has been suggested as a marker of infection [8]. This protein has a role in the innate immune response. It binds to lipopolysaccharide and thereafter brings lipopolysaccharide to the CD14 receptors on the monocyte-macrophage cell lineage. CD14 receptors then interact with Toll-like receptor-4, initiating cytokine production [9,10]. LBP has a longer half-life than the cytokines it induces [11]. These aspects make it interesting to evaluate LBP in infection and sepsis.

High levels of IL-6 have been associated with severe inflammation and sepsis [12-15]. IL-6 has a central role in inducing the synthesis of acute-phase proteins such as CRP and LBP [16]. IL-6 elevations are seen earlier than the elevation of the aforementioned acute-phase proteins. This makes IL-6 an interesting molecule to evaluate in the early phase of infection and sepsis.

An ideal marker of infection and sepsis should have several qualities. A high sensitivity will ensure that all infected patients have a positive result, and a high specificity is required to avoid that patients without infection are diagnosed as having an infection. Furthermore, it should be possible to analyze the marker in a rapid assay with high accuracy.

We have previously shown that CRP and IL-6 are better markers of infection and severity of infection than soluble hemoglobin scavenger receptor (sCD163) in a population of patients admitted to a Department of Internal Medicine [17]. In the present study we examined and compared the performance of CRP and IL-6 with that of PCT and LBP in the same population of patients. We used assays that all could be performed in a routine Department of Clinical Biochemistry.

## Methods

### Patients

Patients were included in a prospective manner in the period January–May 2003. The patients were referred by a general practitioner or were admitted from the Emergency Room. Odense University Hospital is a 1,200 bed health care facility serving a local population of approximately 185,000 inhabitants. The study setting was a Department of Internal Medicine covering the specialties of infectious diseases, rheumatology, pulmonary medicine and general internal medicine. Inclusion criteria for study were suspected diagnosis of infection as judged by the referring physician and blood cultures drawn at the time of admission. The exclusion criteria were age <18 years, earlier participation in the study or prior hospitalization within seven days before admission. Plasma for later analyses of PCT, LBP and IL-6 were drawn immediately after admission. The samples were processed and frozen at -80°C within 1.5 hours. Sampling was performed before any antibiotic treatment was started at the hospital. The patients received a standard of care according to the departmental guidelines. The project protocol was approved by the Ethics Committee of Fyns and Vejle Counties. Informed consent was obtained from all patients or their close relatives.

Baseline characteristics, demographic data, biochemical parameters, SIRS criteria and severity score were obtained at the time of inclusion. Severity was assessed with the Sepsis-related Organ Failure Assessment score [18]. Comorbidity was assessed with the Charlson Index [19]. Patients were classified at the time of admission according to the SIRS criteria [3]. Severe sepsis was defined as the presence of sepsis and one or several of the following indices of organ dysfunction: Glasgow coma scale ≤14, PaO_2 _≤9.75 kPa, oxygen saturation ≤92%, PaO_2_/FiO_2 _≤250, systolic blood pressure ≤90 mmHg, systolic blood pressure fall ≥40 mmHg from baseline, pH ≤7.3, lactate ≥2.5 mmol/l, creatinine ≥177 μmol/l, 100% increase of creatinine in patients with known kidney disease, oliguria ≤30 ml/hour in >3 hours or ≤0.7 l/24 hours, prothrombin time ≤0.6 (reference: 0.70–1.30), platelets ≤100 × 10^9^/l, bilirubin ≥43 μmol/l, and paralytic ileus. Septic shock was defined as hypotension persisting despite adequate fluid resuscitation for at least 1 hour. If a patient had any comorbidity that could more probably explain one or more of the criteria for organ dysfunction stated earlier, the patient could not be categorized as having severe sepsis.

Infection was categorized according to the following definitions: culture/microscopy of a pathogen from a clinical focus; positive urine dip test in the presence of dysuria symptoms; chest X-ray-verified pneumonia with no identified pathogen; infection documented with another imaging technique with no identified pathogen; obvious clinical infection (for instance, erysipelas, wound infection); and identification of a pathogen by serology or PCR. The classification of the status of infection was made by a single physician who was blinded to all biochemical laboratory results. The patients were divided into the following groups for the subsequent statistical analyses: noninfected patients without SIRS, noninfected patients with SIRS, infected patients without SIRS, patients with sepsis, and patients with severe sepsis/septic shock. Patients who could not be classified were excluded from the analyses.

### Laboratory assays

PCT was measured with a time-resolved amplified cryptate emission technology assay (Kryptor PCT^®^; Brahms, Hennigsdorf, Germany). The functional assay sensitivity was 0.06 ng/ml. LBP and IL-6 were measured with a chemiluminescent immunometric assay (Immulite-1000^®^; DPC, Los Angeles, CA, USA). The detection limit of LBP was 0.2 μg/ml. The detection limit of IL-6 was 2 pg/ml. CRP was measured with an immunoturbidometric principle (Modular P^®^; Hitachi, Tokyo, Japan). White blood cells and neutrophils were counted on a Sysmex SE 9000^® ^(TOA^®^, Kobe, Japan). PCT, LBP and IL-6 measurements were carried out in duplicate and the mean values were used for analyses.

### Statistical analysis

Data are presented as medians, interquartile ranges and means ± standard deviation. Significance testing was carried out using the Kruskal–Wallis test. A two-tailed *P *value < 0.05 was considered statistically significant. Receiver-operator characteristic (ROC) curves and the area under the curve (AUC) were determined for PCT, LBP, IL-6, CRP, white blood cells and neutrophils. AUC values are reported with the 95% confidence interval (95% CI). The method described by DeLong and colleagues was used as the significance test for ROC and AUC comparison [20]. Sensitivities, specificities, positive predictive values and negative predictive values were calculated from cross-tabulations. The positive likelihood ratio and negative likelihood ratio were also reported.

Prior to the study we chose the following cut off levels for reporting sensitivities, specificities, positive predictive values, negative predictive values, positive likelihood ratios and negative likelihood ratios: PCT, 0.1 ng/ml, 0.25 ng/ml and 0.5 ng/ml; LBP, 20 μg/ml and 40 μg/ml; CRP, 50 mg/l and 100 mg/l; and IL-6, 25 pg/ml and 50 pg/ml. We also planned to report cut off levels, specificities, positive predictive values, negative predictive values, positive likelihood ratios and negative likelihood ratios with sensitivities of approximately 80%. We intended to compare the test performance by comparing the AUCs and by comparing the specificities when the sensitivity was approximately 80%. The Spearman rank correlation test was used to determine correlations. At the time of the study the Department of Clinical Biochemistry did not report levels of CRP below 10 mg/l; CRP measurements below 10 mg/l were therefore assigned a value of 10 mg/l for calculations. The detection limit of our method for IL-6 measurements was 2 pg/ml; IL-6 measurements below 2 pg/ml were therefore assigned a value of 2 pg/ml for calculations. Statistical calculations were performed in STATA 8 (STATA Corporation^®^, College Station, TX, USA).

## Results

### Patient characteristics

One hundred and ninety-four adult patients were included in our study. The patients were divided according to our definitions into the following groups: 48 noninfected patients without SIRS, 19 noninfected patients with SIRS, 32 infected patients without SIRS, 47 patients with sepsis, and 27 patients with severe sepsis or septic shock. Only one patient had septic shock. This patient was included in the severe sepsis group. Twenty-one patients could not be classified and were excluded from analyses. Fifteen (22.4%) of the noninfected patients were treated with prednisolone and one treated with methotrexate at the time of admission. Fifteen (14.2%) of the infected patients were treated with prednisolone at the time of admission. The baseline characteristics, the outcome, and the microbiology and focus of infection are presented in Tables 1, 2, 3. The final diagnoses of the noninfected patients are described in Table 4.

### Levels of PCT, LBP, IL-6 and CRP

The levels of PCT, LBP, IL-6 and CRP were statistically significantly higher among all infected patients compared with noninfected patients (*P *< 0.001) (Table 5). There was a small increase in PCT levels from the group of noninfected patients to the group of infected patients without SIRS and to the group of sepsis patients. Patients with severe sepsis had almost 10-fold higher levels of PCT compared with patients with sepsis. Levels of LBP, IL-6 and CRP increased progressively with increasing severity of infection/sepsis.

Diagnostic performance of PCT, LBP, IL-6, CRP, white blood cell count and neutrophils in diagnosing infection, sepsis and severe sepsis

**Figure 1:**
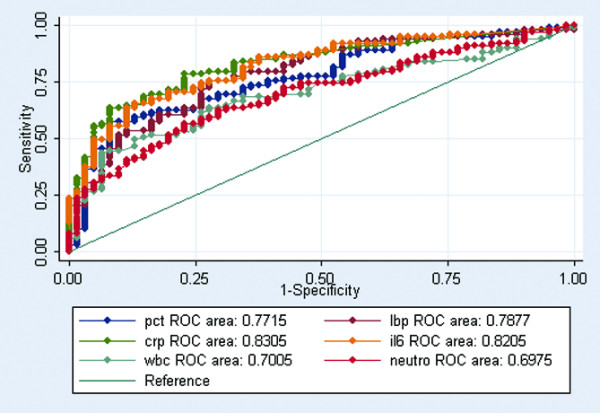
**ROC curves comparing inflammatory markers discriminating abilities between noninfected patients and all infected patients (P < 0.05).** Receiver-operating characteristic (ROC) curves comparing procalcitonin (pct), lipopolysaccharide-binding protein (lbp), C-reactive protein (crp), IL-6 (il6), white blood cell (wbc) and neutrophil (neutro) discriminating abilities between noninfected patients and all infected patients (*P *< 0.05).

In a ROC analysis to distinguish between noninfected patients and infected patients, CRP and IL-6 had the highest AUC values of 0.83 (95% CI 0.76–0.89) and 0.82 (95% CI 0.75–0.88) (Figure 1). PCT performed with an AUC of 0.77 (95% CI 0.69–0.84) and LBP with an AUC of 0.78 (95% CI 0.71–0.85) (Figure 1). Using a cut off level of 30 mg/l, CRP had a sensitivity of 80.2% and a specificity of 62.7% in diagnosing infection (Table 6). Using a cut off level of 16.3 pg/ml, IL-6 had a sensitivity of 79.2% and a specificity of 64.2% in diagnosing infection (Table 6).

**Figure 2:**
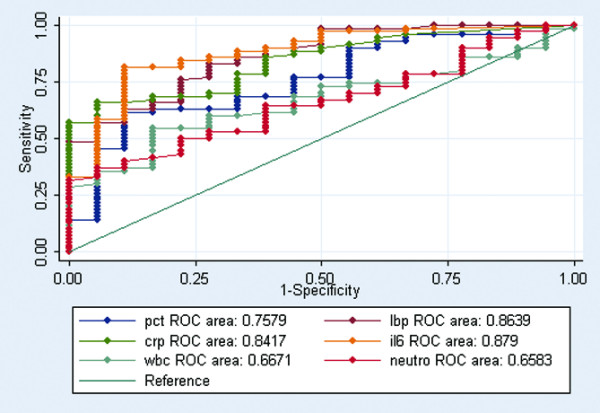
**ROC curves comparing inflammatory markers discriminating abilities between systemic inflammatory response syndrome and sepsis(P 2 0.01).** Receiver-operating characteristic (ROC) curves comparing procalcitonin (pct), lipopolysaccharide-binding protein (lbp), C-reactive protein (crp), IL-6 (il6), white blood cell (wbc) and neutrophil (neutro) discriminating abilities between systemic inflammatory response syndrome (SIRS) (noninfected with SIRS) and sepsis (sepsis and severe sepsis) (*P *< 0.01).

In a ROC analysis to distinguish between patients with noninfectious SIRS and patients with sepsis/severe sepsis, IL-6, LBP and CRP had an AUC of 0.87 (95% CI 0.78–0.96), 0.86 (95% CI 0.77–0.95) and 0.84 (95% CI 0.75–0.92), respectively (Figure 2). PCT had an AUC of 0.75 (95% CI 0.63–0.87) (Figure 2). Using a cut off level of 25 pg/ml, IL-6 had a sensitivity of 81.1% and a specificity of 78.9% in diagnosing sepsis/severe sepsis (Table 7). Using a cut-off level of 20 μg/ml, LBP had a sensitivity of 81.0% and a specificity of 68.4% in diagnosing sepsis/severe sepsis (Table 7). Using a cut off level of 38 mg/l, CRP had a sensitivity of 79.7% and a specificity of 57.9% in diagnosing sepsis/severe sepsis (Table 7).

**Figure 3:**
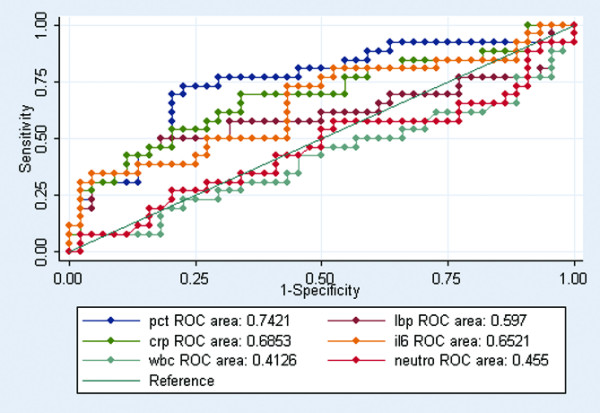
**ROC curves comparing inflammatory markers discriminating abilities between sepsis and severe sepsis (P 2 0.01).** Receiver-operating characteristic (ROC) curves comparing procalcitonin (pct), lipopolysaccharide-binding protein (lbp), C-reactive protein (crp), IL-6 (il6), white blood cell (wbc) and neutrophil (neutro) discriminating abilities between sepsis and severe sepsis (*P *< 0.01).

In a ROC analysis to distinguish between patients with sepsis and patients with severe sepsis, PCT performed best with an AUC of 0.74 (95% CI 0.61–0.87) (Figure 3).

### Correlations between the examined markers

A strong correlation was found between LBP and CRP (*r *= 0.842, *P *< 0.0001) and a weaker correlation was found between LBP and IL-6 (*r *= 0.568, *P *< 0.0001). Weak correlations were found between PCT, CRP and IL-6.

## Discussion

The patients included in this study were elderly patients with a burden of comorbidity representative of medical patients admitted to a Department of Internal Medicine. The mortality among the infected patients was only 3.8% and the severity of sepsis was low as judged by the Sepsis-related Organ Failure Assessment score. Our patients therefore had relatively mild disease compared with patients included in most other diagnostic test studies focusing on infection and sepsis [21-27]. This study therefore adds valuable information on markers of sepsis.

If new diagnostic markers are considered for introduction in nonintensive care patients or patients with less severe disease it is important that they are validated in the relevant population. Our study population was well characterized and the study had a prospective design. We avoided workup bias by blinding the physician scoring the infection status from all biochemical laboratory results. We tried to minimize spectrum bias by using relatively liberal inclusion criteria. We used a sensitive PCT assay that made it possible also to determine PCT levels between 0.06 ng/ml and 0.5 ng/ml. This made it possible to examine lower cut off levels for PCT, which was important since we studied less ill patients where we could expect lower PCT levels than those reported among patients in intensive care units. Our definition of infection did not exclude patients with viral infection.

There were eight confirmed cases with viral infection, and it is possible that some patients where no pathogen was identified had viral infection. In our opinion this reflects the clinical reality, where often no etiological agent is identified despite thorough clinical and laboratory investigations. A drawback in this study design is the possibility of imperfect gold standard bias. If the test and imperfect gold standard are independent we can expect that the sensitivity and specificity of the test will be underestimated. Because of the risk of imperfect gold standard bias, we also analyzed the diagnostic test abilities of our candidate markers, after having excluded all patients without microbiological proven infection. The results of these analyses did not, however, lead to a different conclusion on the utility of the candidate markers (data not shown).

The biological role of PCT has not yet been clarified [28]. Some studies have suggested PCT to be a secondary mediator involved in the immunopathogenesis in sepsis. Administration of PCT to septic hamsters increased mortality, and the neutralization of PCT with antiserum to septic hamsters reduced mortality [29]. This suggests that the highest levels of PCT may be seen in severe sepsis with high mortality. The low levels of PCT in our study probably reflect that we were focusing on a population with relatively mild disease. It is possible that elevated levels of PCT are mainly seen in patients with severe sepsis with high Sepsis-related Organ Failure Assessment scores and in patients with septic shock.

Several studies have focused on the diagnostic test abilities of PCT to diagnose sepsis in patients requiring intensive care [21-27]. These studies found sensitivities between 65% and 97% and specificities between 48% and 94%. Three of these studies found PCT to be a better sepsis marker than CRP [22,24,25]. In the study by Ugarte and colleagues, however, CRP performed better than PCT [21]. Also, PCT and CRP performed equally well in the study by Suprin and colleagues [23]. Few studies have been conducted in patients not admitted to intensive care units. These studies have found sensitivities between 24% and 74% and specificities between 70% and 94% [30-34]. PCT was not a better marker of bacterial infection than CRP in the study by Chan and colleagues [32]. PCT had a lower sensitivity and a higher specificity while CRP had a higher sensitivity and a lower specificity in the study by Stucker and colleagues [34].

These studies mentioned used less sensitive methods for PCT analyses than in the present study. In our study PCT performed poorer than CRP, IL-6 and LBP in diagnosing infection and in discriminating between noninfectious SIRS and sepsis/severe sepsis. In contrast, PCT performed best in a ROC analysis distinguishing between patients with sepsis and patients with severe sepsis, supporting other findings of PCT being a marker reflecting the severity of sepsis [21,22].

LBP has a central role in the early activation of the innate immune response [9]. LBP, like CRP, is an acute-phase protein produced in the liver. Although the function of LBP is to bind lipopolysaccharide from Gram-negative bacteria, elevated levels of LBP are also seen in Gram-positive infections [35]. This is an important observation if LBP is considered as a marker for both Gram-negative infection and Gram-positive infection. We found a strong correlation between LBP and CRP suggesting a common activation or a common pathway for these acute phase proteins.

A few studies have investigated LBP levels in infection and sepsis [11,35-39]. To our knowledge only three studies have focused on LBP diagnostic test abilities in severe infections [37-39]. The study by Oude Nijhuis and colleagues found a sensitivity of 100% and a specificity of 92% in diagnosing Gram-negative bacteremia in cancer patients with neutropenia [37]. They used a high cut off level (46.3 μg/ml) for LBP. The study by Prucha and colleagues found a sensitivity of 50% and a specificity of 74.2% in discriminating between noninfectious SIRS and sepsis, in a cohort of patients requiring intensive care [38]. The study by Pavcnik-Arnol and colleagues found a sensitivity of 97% and a specificity of 70% in diagnosing sepsis in critically ill children [39]. In their study LBP performed equally compared with CRP, but was superior to IL-6 and PCT. Our data suggest that LBP performs better than PCT as a diagnostic marker for infection and sepsis.

A correlation between IL-6 levels and the severity/mortality of sepsis has been observed in several studies [13-15]. Sensitivities between 65.0% and 86.0% and specificities between 54.0% and 79.0% have been found in diagnosing sepsis [24-26,40]. In three of these studies PCT was superior to IL-6 [24,26,40]. This is contrary to our data, which suggest that IL-6 is superior to PCT as a diagnostic marker for infection and sepsis.

Several studies have focused on the diagnostic test abilities of CRP in diagnosing infection and/or sepsis [21,23-25,30,32,34,41,42]. These studies found sensitivities between 67.2% and 94.3% and specificities between 33.0% and 93.9%. In our study CRP performed better than PCT as a diagnostic marker for infection and sepsis.

A diagnostic marker of any disease should provide the clinician with useful information to increase the likelihood of diagnosing either if the disease is actually present or if the disease is in fact absent. Because prompt and effective antibiotic treatment is crucial in the treatment of patients with infections and sepsis, any new potential diagnostic marker of infection should have a high sensitivity, so as many as possible of the infected patients are diagnosed as early as possible. This may lead to some overuse of antibiotics because of a lower specificity, but in terms of consequence for the individual patient we consider this to be a lesser concern than withholding antibiotics from the infected patient.

Our study data suggest that LBP (cut off level 20 μg/ml), CRP (cut off level 30 mg/l) and IL-6 (cut off level 16.3 pg/ml) are comparable in terms of their diagnostic abilities in diagnosing infection. A high sensitivity and a high specificity are also important qualities that should be required from any new potential diagnostic marker distinguishing between SIRS without infection and sepsis. Our study data suggest that IL-6 with a cut off level of 25 pg/ml has the best diagnostic abilities in diagnosing sepsis. With this cut off level, IL-6 has a sensitivity and a specificity of approximately 80%. An effective new potential diagnostic marker could also have qualities in identifying noninfected patients with or without SIRS. This would require a high specificity. Our study data suggest that CRP (cut off level 100 mg/l) and IL-6 (cut off level 50 pg/ml) have the best qualities in identifying the noninfected patients. With these cut off levels CRP and IL-6 have sensitivities higher than 58% and specificities greater than 88% in diagnosing infection.

## Conclusion

Data from earlier studies and from our study suggest that the markers examined in the present study can have different test qualities depending on the study population. It is important to look separately at the test qualities on an intensive care unit population dominated by severe sepsis/septic shock, and those on an internal medicine population, dominated by the milder end of the sepsis spectrum. Our data suggest that PCT does not have a diagnostic role in patients with mild infection/sepsis admitted to a Department of Internal Medicine. IL-6, CRP and LBP appear to be of equal value as diagnostic infection markers in our study. They performed better than PCT, but are all relatively poor markers for infection with sensitivity/specificity below 80% with the chosen cut-off levels. IL-6, LBP and CRP appear to be superior as diagnostic sepsis markers compared with PCT. Only IL-6 reached a sensitivity and specificity of approximately 80% in diagnosing sepsis with a cut-off level of 25 pg/ml.

## Key messages

• 	In a cohort of patients with less severe community-acquired infections, CRP, IL-6 and LBP appear to be superior to PCT as diagnostic markers for infection.

• 	In a cohort of patients with less severe community-acquired infections, CRP, IL-6 and LBP appear to be superior to PCT as diagnostic markers for sepsis.

• 	PCT appears to be a marker for the severity of sepsis at the time of admission.

## Abbreviations

AUC = area under the curve; 95% CI = 95% confidence interval; CRP = C-reactive protein; IL = interleukin; LBP = lipopolysaccharide-binding protein; PCR = polymerase chain reaction; PCT = procalcitonin; ROC = receiver-operating characteristic; SIRS = systemic inflammatory response syndrome.

## Competing interests

The authors declare they have no competing interests.

## Authors' contributions

SG planned the study, wrote the protocol, collected and analyzed the data, and wrote the report. OGK was responsible for PCT, IL-6 and LBP analyses. SSP and CP were involved in planning the study and were involved in the practical clinical aspects.

## Figures and Tables

**Table 1:** Baseline characteristics of the patients

Variable	Noninfected without SIRS (*n *= 48)	Noninfected with SIRS (*n *= 19)	Infection without SIRS (*n *= 32)	Sepsis (*n *= 47)	Severe sepsis (*n *= 27)
Male	16 (33.3)	7 (36.8)	18 (56.3)	20 (42.6)	18 (66.7)
Female	32 (66.6)	12 (63.2)	14 (43.7)	27 (57.4)	9 (33.3)
Age	68.4 ± 18	64.4 ± 14.6	60.8 ± 16.6	60.4 ± 19.9	66.4 ± 17.8
SOFA score	1.3 ± 1.1	1.7 ± 1.1	1.6 ± 1.5	1.6 ± 1.2	3.0 ± 1.9
Charlson Index of comorbidity	1.5 ± 1.3	1.9 ± 1.2	1.3 ± 1.3	1.1 ± 1.3	1.2 ± 1.3
Hemoglobin (mmol/l)	7.9 ± 1.0	8.5 ± 1.1	8.2 ± 1.2	8.2 ± 1.2	8.2 ± 1.1
Platelet count (10^9^/l)	291 ± 115.5	283 ± 89.1	325 ± 210.6	254 ± 107.3	268 ± 184.4
Bilirubin (μmol/l)	9.3 ± 6.7	9.5 ± 7.6	21.9 ± 36.6	10.6 ± 6.8	13.6 ± 5.5
Prothrombin time	1.0 ± 0.3	0.9 ± 0.3	0.9 ± 0.4	0.9 ± 0.3	0.9 ± 0.3
Creatinine (μmol/l)	96.9 ± 27.1	96.1 ± 28.5	100.6 ± 31.2	100.4 ± 31.7	140.3 ± 79.5

Data are presented as the absolute number (%) or the mean ± standard deviation. SIRS, systemic inflammatory response syndrome; SOFA, Sepsis-related Organ Failure Assessment.

**Table 2:** Outcome of the patients

Variable	Noninfected without SIRS (*n *= 48)	Noninfected with SIRS (*n *= 19)	Infection without SIRS (*n *= 32)	Sepsis (*n *= 47)	Severe sepsis (*n *= 27)
Hospitalization (days)	8.4 ± 6.7	8.7 ± 7.8	10.3 ± 11.5	7.8 ± 6.7	10.8 ± 10.5
Mortality on day 28	2 (4.2)	4 (21.1)	0	0	4 (14.8)

Data are presented as the absolute number (%) or the mean ± standard deviation. SIRS, systemic inflammatory response syndrome.

**Table 3:** Microbiological and infection characteristics of the patients

Variable	Infection without SIRS (*n *= 32)	Sepsis (*n *= 47)	Severe sepsis (*n *= 27)
Assessment of infection (*n*)			
Gram-positive bacteria	6	12	10
Gram-negative bacteria	7	10	7
Other bacteria	0	2^a^	0
Bacteremia	1	4	7
Virus	3^b^	4^c^	1^d^
Chest X-ray-verified pneumonia^e^	9	13	7
Radiological evidence^f^	0	1	0
Obvious clinical infection^g^	7	5	2
Focus of infection (*n*)			
Upper respiratory tract infection	1	0	1
Lower respiratory tract infection	12	25	15
Endocarditis	1	0	1
Gastroenteritis	5	1	0
Pyelonephritis	2	2	1
Cystitis	0	3	3
Skin/soft tissue infection	1	4	3
Bone/joints	2	1	0
Other	8	11	3

SIRS, systemic inflammatory response syndrome. ^a^*Mycoplasma pneumoniae *(*n *= 2). ^b^Epstein–Barr virus (*n *= 1), influenzae A virus (*n *= 2). ^c^Epstein–Barr virus (*n *= 2), influenza A virus (*n *= 2). ^d^Puumala virus (*n *= 1). ^e^Chest X-ray-verified pneumonia with no identified pathogen. ^f^Infection documented by imaging techniques (other than Chest X-ray) with no identified pathogen. ^g^Clinical infection (i.e. erysipelas, wound infection).

**Table 4:** Diagnoses of the non-infected patients (*n *= 67)

Diagnosis	Number of patients
Central nervous system disease	5
Cardiovascular disease	10
Respiratory disease	33^a^
Gastroenterological disease	2
Hematological disease	2
Malignant disease	4
Rheumatological disease	8
Renal disease	1
Dehydration	2

^a^Chronic obstructive pulmonary disease (*n *= 22).

**Table 5:** Levels of procalcitonin, lipopolysaccharide-binding protein, C-reactive protein, IL-6, white blood cells and neutrophils in different groups

Variable^a^	Noninfected without SIRS (*n *= 48)	Noninfected with SIRS (*n *= 19)	Infection without SIRS (*n *= 32)	Sepsis (*n *= 47)	Severe sepsis (*n *= 27)
Procalcitonin (ng/ml)					
Median	0.07	0.09	0.16	0.2	1.9
Interquartile range	0.05–0.11	0.05–0.14	0.07–0.34	0.08–0.65	0.22–14.6
Lipopolysaccharide-binding protein (μg/ml)					
Median	16.3	16.4	27.4	33.5	40.4
Interquartile range	12.6–25.3	11.3–26.5	18.3–41.2	25.0–43.2	18.0–63.6
C-reactive protein (mg/l)					
Median	18.0	19.0	122.0	120.0	217.0
Interquartile range	10.0–38.0	10.0–65.0	54.0–215.0	41.0–190.0	78.0–414.0
IL-6 (pg/ml)					
Median	8.7	9.8	20.6	72.6	199.3
Interquartile range	3.2–20.7	2.0–23.7	9.8–99.4	25.9–274.5	67.5–2833.0
White blood cells (10^9^/l)					
Median	7.8	9.5	9.5	13.0	12.2
Interquartile range	6.7–9.2	7.8–12.1	7.7–11.9	9.2–17.1	7.0–17.5
Neutrophils (10^9^/l)					
Median	5.9	7.6	7.1	10.1	10.3
Interquartile range	4.6–6.9	6.2–9.8	5.1–9.7	7.1–14.8	5.5–15.4

SIRS, systemic inflammatory response syndrome. ^a^*P *< 0.001 by the Kruskal–Wallis test.

**Table 6:** Sensitivity, specificity, positive predictive value, negative predictive value, positive likelihood ratio and negative likelihood ratio of inflammatory markers in diagnosing infection

Variable	Cut-off level	Sensitivity (%)	Specificity (%)	Positive predictive value (%)	Negative predictive value (%)	Positive likelihood ratio	Negative likelihood ratio
Procalcitonin	0.075 ng/ml	80.2	47.8	70.8	60.4	1.5	0.41
	0.1 ng/ml	71.6	62.7	75.3	58.3	1.9	0.45
	0.25 ng/ml	48.1	89.5	87.9	52.2	4.6	0.58
	0.5 ng/ml	37.7	95.5	93.0	49.2	8.4	0.65
Lipopolysaccharide-binding protein	20 μg/ml	78.3	64.2	77.6	65.2	2.2	0.34
	40 μg/ml	37.7	91.0	86.9	48.0	4.2	0.68
C-reactive protein	30 mg/l	80.2	62.7	77.3	66.7	2.2	0.32
	50 mg/l	73.6	74.6	82.1	64.1	2.9	0.35
	100 mg/l	62.3	89.5	90.4	60.0	5.9	0.42
IL-6	16.3 pg/ml	79.2	64.2	77.8	66.2	2.2	0.32
	25 pg/ml	70.8	77.6	83.3	62.6	3.2	0.38
	50 pg/ml	58.5	88.0	88.6	57.3	4.9	0.47

**Table 7:** Sensitivity, specificity, positive predictive value, negative predictive value, positive likelihood ratio and negative likelihood ratio of inflammatory markers in diagnosing sepsis

Variable	Cut-off level	Sensitivity (%)	Specificity (%)	Positive predictive value (%)	Negative predictive value (%)	Positive likelihood ratio	Negative likelihood ratio
Procalcitonin	0.087 ng/ml	79.7	42.1	84.3	34.8	1.4	0.48
	0.1 ng/ml	75.7	52.6	86.2	35.7	1.6	0.46
	0.25 ng/ml	55.0	89.5	95.4	34.0	5.2	0.5
	0.5 ng/ml	45.9	89.5	94.4	29.8	4.4	0.6
Lipopolysaccharide-binding protein	20 μg/ml	81.0	68.4	90.9	48.2	2.6	0.28
	40 μg/ml	41.9	100.0	100.0	30.7		0.58
C-reactive protein	38 mg/l	79.7	57.9	88.1	42.3	1.9	0.35
	50 mg/l	71.6	63.2	88.3	36.4	1.9	0.45
	100 mg/l	63.5	94.7	97.9	40.0	11.9	0.39
IL-6	25 pg/ml	81.1	78.9	93.8	51.7	3.8	0.24
	50 pg/ml	70.3	89.5	96.3	43.6	6.7	0.33
